# Retrospective German claims data study on initial treatment of bladder carcinoma (BCa) by transurethral bladder resection (TURB): a comparative analysis of costs using standard white light- (WL-) vs. blue light- (BL-) TURB

**DOI:** 10.1007/s00345-020-03587-0

**Published:** 2021-02-10

**Authors:** Tilman Todenhöfer, Moritz Maas, Miriam Ketz, Nils Kossack, Christiane Colling, Bryan Qvick, Arnulf Stenzl

**Affiliations:** 1 Department of Urology, University of Tuebingen, Hoppe-Seyler-Straße 3, 72076 Tübingen, Germany; 2 Studienpraxis Urologie, Steinengrabenstr. 17, 72622 Nürtingen, Germany; 3 DtoD – Data to Decision – AG, Heimhuder Straße 52, 20148 Hamburg, Germany; 4 WIG2 GmbH, Scientific Institute for Health Economics and Health System Research, Markt 8, 04109 Leipzig, Germany; 5 Ipsen Pharma GmbH, Einsteinstr. 174, 81677 Munich, Germany

**Keywords:** Urothelial cancer, Transurethral bladder resection, Hexaminolevulinate, Photodynamic diagnosis, Retrospective health service research, German claims data

## Abstract

**Purpose:**

Photodynamic diagnosis using hexaminolevulinate (HAL)—guided BL-TURB may reduce the recurrence risk in non-muscle invasive BCa compared to standard WL-TURB due to more sensitive tumor detection. The impact of the initial use of WL- vs. BL-TURB on follow-up costs was evaluated in this real-world data analysis.

**Methods:**

Anonymous claims data of German statutory health insurances (GKV) from 2011 to 2016 were analyzed in a primary and adjusted study population. Selection criteria included five quarters before enrolment, one index quarter (InQ) of initial TURB and BCa diagnosis, either within two years for the primary analysis or within four years for the adjusted analysis, and a follow-up period (FU) of either eleven or three quarters, respectively.

**Results:**

In the primary analysis (*n* = 2331), cystectomy was identified as an important cost driver masking potential differences between cohorts. Therefore, patients undergoing cystectomy (InQ + FU) were excluded from the adjusted study population of *n* = 4541 patients (WL: 79%; BL: 21%). Mean total costs of BL-TURB were initially comparable to WL-TURB (WL: EUR 4534 vs. BL: EUR 4543) and tended to be lower compared to WL-TURB in the first two quarters of FU. After one year (3rd FU quarter), costs equalized. Considering total FU, mean costs of BL-TURB were significantly lower compared to WL-TURB (WL: EUR 7073 vs BL: EUR 6431; *p* = 0.045).

**Conclusion:**

This retrospective analysis of healthcare claims data highlights the comparability of costs between BL-TURB and WL-TURB.

## Introduction

Bladder cancer (BCa) is among the ten most common cancers worldwide, with every third new case occurring in Europe [1], and also one of the most cost-consuming cancer diseases [2]. Its incidence is higher for men than for women and increases with age [3, 4]. The most common histological form, accounting for approximately 70% of primary diagnosed bladder tumors, is non-muscle-invasive BCa [1, 3].

Patients with suspected BCa are visually examined by white light (WL) cystoscopy as a standard diagnostic procedure [3, 5, 6]. To confirm the diagnosis and establish the tumor state, a transurethral resection of the bladder (TURB) is routinely performed, which for non-muscle-invasive tumors also constitutes the initial treatment option, generally followed by immediate intravesical instillation of chemotherapy [3, 6]. Only 10–20% of non-muscle-invasive tumors progress to muscle-invasive tumors, but 50–70% of non-muscle-invasive tumors will recur, highlighting the need for optimal initial detection and treatment to ensure optimal prognosis [1]. Hexaminolevulinate (HAL) was approved in the EU and the US for assessment of non-muscle-invasive BCa by photodynamic diagnosis. After instillation of HAL into the bladder, photoactive porphyrin accumulates in neoplastic cells and facilitates their detection by emitting red fluorescence during cystoscopy with blue light (BL) [7]. The detection rate of tumors is improved by BL-cystoscopy compared to WL-cystoscopy by 10–20% for non-invasive papillary carcinoma and by up to 40% for carcinoma in situ [8–11]. Furthermore, the recurrence rate is reduced [10, 12–14] and recurrence-free survival is prolonged [13, 15] when using BL- compared to WL-cystoscopy. The impact on progression remains unclear [16–19] and depends on the criteria used to define progression [18].

Due to the high recurrence risk of BCa, patients require continuous monitoring. The quality of the initial TURB, however, impacts prognosis and thereby also treatment costs. Models evaluating cost-effectiveness of BL- compared to WL-TURB predict increased quality-adjusted life years and lower long-term costs for BL-TURB despite BL-TURB being more expensive than WL-TURB [20–23]. So far there is only limited information available concerning initial use of WL- or BL-TURB. Using German claims data, the objective of this study was to analyze the real-world impact on costs in case of either WL- or BL-TURB applied as initial treatment in patients with BCa.

## Materials and methods

### Data source

For this retrospective analysis, routine healthcare claims data from more than 60 German statutory health insurances (GKV, *Gesetzliche Krankenversicherung*) were used [24]. The sample comprised more than 4.5 Million individuals, GKV-insured at least one day between 2011-01-01 and 2016-12-31 and was representative concerning age, gender and morbidity in Germany. As anonymized and pseudonymized healthcare claims data were evaluated, the study was exempt from ethical approval.

### Study population

**Fig. 1:**
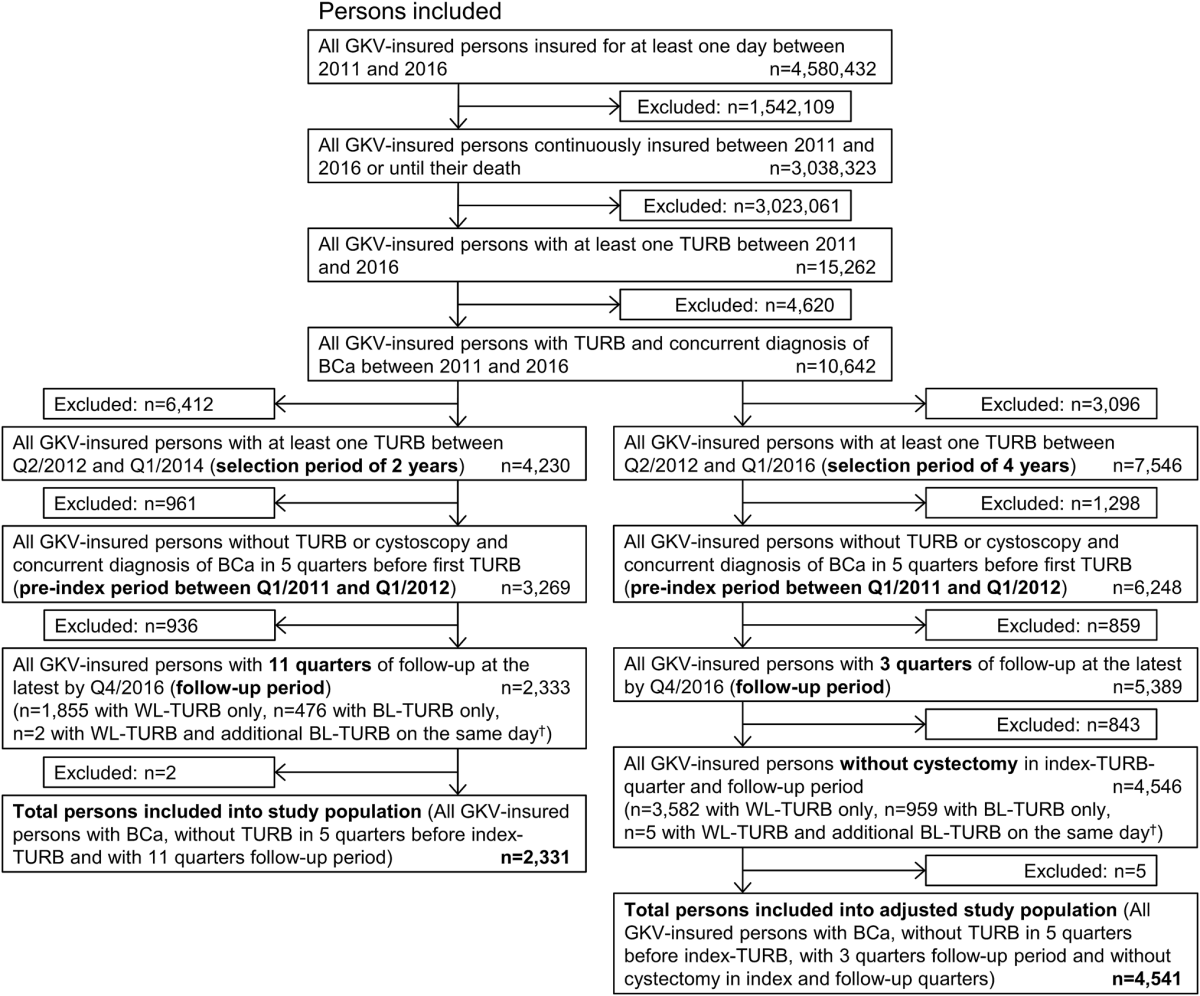
Selection of study population (left) and adjusted study population (right). Inclusion criteria were applied stepwise. Diagnosis of bladder cancer (BCa) was based on International Classification of Diseases, 10th Revision, German Modification (ICD-10-GM): C67, malignant neoplasm of bladder or D09.0, carcinoma in situ of the bladder; inpatient or outpatient confirmed. TURB, cystoscopy and cystectomy were based on German Operations and Procedures Key (OPS Code): 5-573.40 (WL-TURB), 5-573.41/5-573.4x (BL-TURB), inpatient (OPS code 5-573.2 for transurethral excision was not included); 1-661/1-663/1-693.2 (cystoscopy), inpatient or outpatient; 5-575 (partial cystectomy), 5-576 (simple/radical cystectomy), inpatient. ^†^*n* < 5 excluded ensuring statistical anonymity.

The step-by-step selection process to generate the study population is shown in Fig. 1. The total study population was adjusted for further analysis of costs as indicated.

### Study time periods

The primary analysis included patients whose index quarter (InQ) comprising initial TURB and concurrent diagnosis of BCa fell in the selection period of two years between Q2/2012 and Q1/2014. A pre-index period without any TURB or cystoscopy before first index TURB included five quarters, from Q1/2011 until Q1/2012. The follow-up period (FU) lasted eleven quarters, ending latest Q4/2016. Each patient’s individual study period was determined by the index date.

For the analysis of the adjusted study population collecting patients without cystectomy, the FU was shortened to three quarters ending latest Q4/2016. The main focus was on the first FU quarters to detect possible direct cost effects after the initial TURB. The pre-index period remained unchanged, resulting in an extended selection period of four years between Q2/2012 and Q1/2016, thus increasing the corresponding study population.

### Treatments

The study population was divided into two cohorts depending on whether they underwent initial WL- or initial BL-TURB. The cohorts were further divided depending on cystectomy for subgroup analysis and for the adjusted study population without cystectomy in InQ and FU.

### Statistics

Descriptive analyses were applied for evaluation, using the *χ*^2^ test for categorical variables and the Wilcoxon rank-sum test for continuous variables. Differences were considered statistically significant if *p *values were < 0.05.

## Results

### Characteristics of the study population

The total study population comprised 2331 patients, 1855 (79.6%) in the WL-cohort and 476 (20.4%) in the BL-cohort; significantly more patients received initial WL- than BL-TURB in the total study population as well as in both male and female subgroups. Likewise, in all persons continuously insured between 2011 and 2016 (*n* = 3,038,323) the incidence rate of initial WL-TURB according to cohort selection (0.06%) was significantly higher than of BL-TURB (0.02%; *p* < 0.001).

The demographic data of the study population are summarized in Table 1. Mean age and gender ratio were comparable between both cohorts, with more than three quarters of patients being men. More than 98% of patients had non-metastatic disease at the time of diagnosis in the InQ (Table 1).

**Table 1:** Demographic data and disease states of bladder cancer of the total study population and per cohort in the index quarter

Characteristics	Study population			
	Total (*N* = 2331)	WL-TURB cohort (*N* = 1855)	BL-TURB cohort (*N* = 476)	*p*^†^
Sex				
Male (%)	79.45	79.78	78.15	0.431
Female (%)	20.55	20.22	21.85	
Age (years)				
Mean ± SD	69.16 ± 10.88	69.48 ± 10.86	67.90 ± 10.86	0.663
Median	71	71	69	
Age group (%)				
0–39	0.90	0.97	0.63	
40–49	4.63	4.20	6.30	
50–59	13.47	13.32	14.08	
60–69	25.48	24.53	29.20	
70–79	38.61	39.46	35.29	
80–89	16.39	17.04	13.87	
90 +	0.51	0.49	0.63	
Stage of bladder cancer according to Coding^‡^ (%)				
N0 and M0	98.93	98.98	98.74	0.655
N1 and M0	0.86	0.81	1.05	0.610
N0 and M1	0.21	0.22	0.21	0.981
N1 and M1	0.00	0.00	0.00	–

^†^WL- vs. BL-cohort. ^‡^The findings are based on the International Classification of Diseases, 10th Revision, German Modification (ICD-10-GM) and do not result from clinical histopathological TNM staging. No lymph node metastases (= N0); no distant metastases (= M0); lymph node metastases (= N1); distant metastases (= M1)

### Cost analysis of the study population

Costs^1^ were calculated from the perspective of a statutory health insurance (Table 2A). In the InQ, the mean total costs of EUR 5687 in the BL-cohort were significantly higher than the mean total costs of EUR 4609 in the WL-cohort (*p* < 0.001), resulting from both higher inpatient and medicinal product costs in the BL-cohort. However, in the FU, mean total costs were not significantly different between both cohorts (WL: EUR 20,442; BL: 20,253; *p* = 0.794). Altogether, total costs over time were EUR 25,940 in the BL-cohort and hence not significantly higher than in the WL-cohort (EUR 25,051; *p* = 0.525).

**Table 2:** Cost tables for study population

2A: Total costs and main cost domains per cohort in index quarter and follow-up period									
	Total (index quarter + 11 quarters follow-up)			Index quarter			Follow-up period (11 quarters)		
Cohort	WL	BL	*p*	WL	BL	*p*	WL	BL	*p*
Study population									
*N*	1,855	476							
Total costs^†^ (€)									
Mean ± SD	25,051 ± 21,607	25,940 ± 42,550	0.525	4609 ± 3974	5687 ± 14,505	< 0.001	20,442 ± 20,480	20,253 ± 31,017	0.794
Median	18,904	17,803		3174	3552		14,261	13,510	
Cost domains (€)									
Outpatient treatment									
Mean ± SD	3977 ± 2754	3875 ± 2110	0.772	402 ± 355	379 ± 270	0.174	3574 ± 2648	3497 ± 1979	0.549
Median	3397	3419		298	309		3032	3065	
Inpatient treatment									
Mean ± SD	15,261 ± 16,054	14,261 ± 14,117	0.215	3857 ± 3821	4309 ± 4388	< 0.001	11,405 ± 15,203	9,953 ± 13,092	0.056
Median	10,262	9419		2322	2811		6726	5329	
Medicinal products									
Mean ± SD	3823 ± 7997	5847 ± 35,455	0.025	286 ± 619	949 ± 12,946	< 0.001	3537 ± 7573	4897 ± 23,822	0.037
Median	2295	2304		110	109		2022	2074	

2B: Total costs per cohort in index quarter and follow-up period for subgroups with and without cystectomy									
	Total (index quarter + 11 quarters follow-up)			Index quarter			Follow-up period (11 quarters)		
Cohort	WL	BL	*p*	WL	BL	*p*	WL	BL	*p*
SUBGROUP WITH CYSTECTOMY^††^									
*N*	284	67							
Total costs (€)									
Mean ± SD	45,947 ± 26,076	58,278 ± 94,333	0.056	7753 ± 6662	14,412 ± 37,038	0.005	38,194 ± 26,729	43,866 ± 60,580	0.719
Median	38,232	42,513		4659	5262		31,744	33,491	
SUBGROUP WITHOUT CYSTECTOMY^††^									
*N*	1,571	409							
Total costs (€)									
Mean ± SD	21,273 ± 18,317	20,642 ± 21,645	0.551	4040 ± 2922	4258 ± 2912	0.179	17,233 ± 17,298	16,384 ± 20,489	0.396
Median	16,221	15,922		3074	3398		12,433	12,016	

^†^Beside the main cost domains outpatient, inpatient, and medicinal products, the cost domains appliances, remedies, and sick pay were also included in total costs but are not shown individually

^††^Cystectomy was based on German Operations and Procedures Key (OPS Code): 5-575 (partial cystectomy), inpatient; 5-576 (simple/radical cystectomy), inpatient

### Subgroup analysis of costs: cystectomy

To investigate the impact of cystectomy in the total study population, a subgroup with cystectomy (*n* = 351) was compared to a subgroup without cystectomy in InQ and FU (*n* = 1980). Cystectomy rates were not significantly different between both cohorts (WL: 15.3%, BL: 14.1%; *p* = 0.502). Regarding total costs over time, subgroup analysis showed that expenses were more than twice as high for patients with cystectomy as for patients without cystectomy (Table 2B). In the cystectomy subgroup, mean total costs were significantly higher in the InQ, resulting from higher inpatient and medicinal product costs. During FU, costs remained on a high, but not significantly different level in both BL- and WL-cohorts. In the subgroup without cystectomy, the mean total costs were considerably lower and similar between the WL- and the BL-cohort in both InQ and FU.

### Cost analysis of the adjusted study population

As cystectomy was identified as an important cost driver contributing greatly to the total costs in both cohorts, an additional cost analysis was performed for an adjusted study population only including patients without cystectomy in InQ and FU. To evaluate any possible direct effects of the first TURB, only the first three quarters of follow-up were included in the analysis. This shortening of the FU resulted in an increased selection period from two to four years yielding a higher number of patients included.

**Fig. 2:**
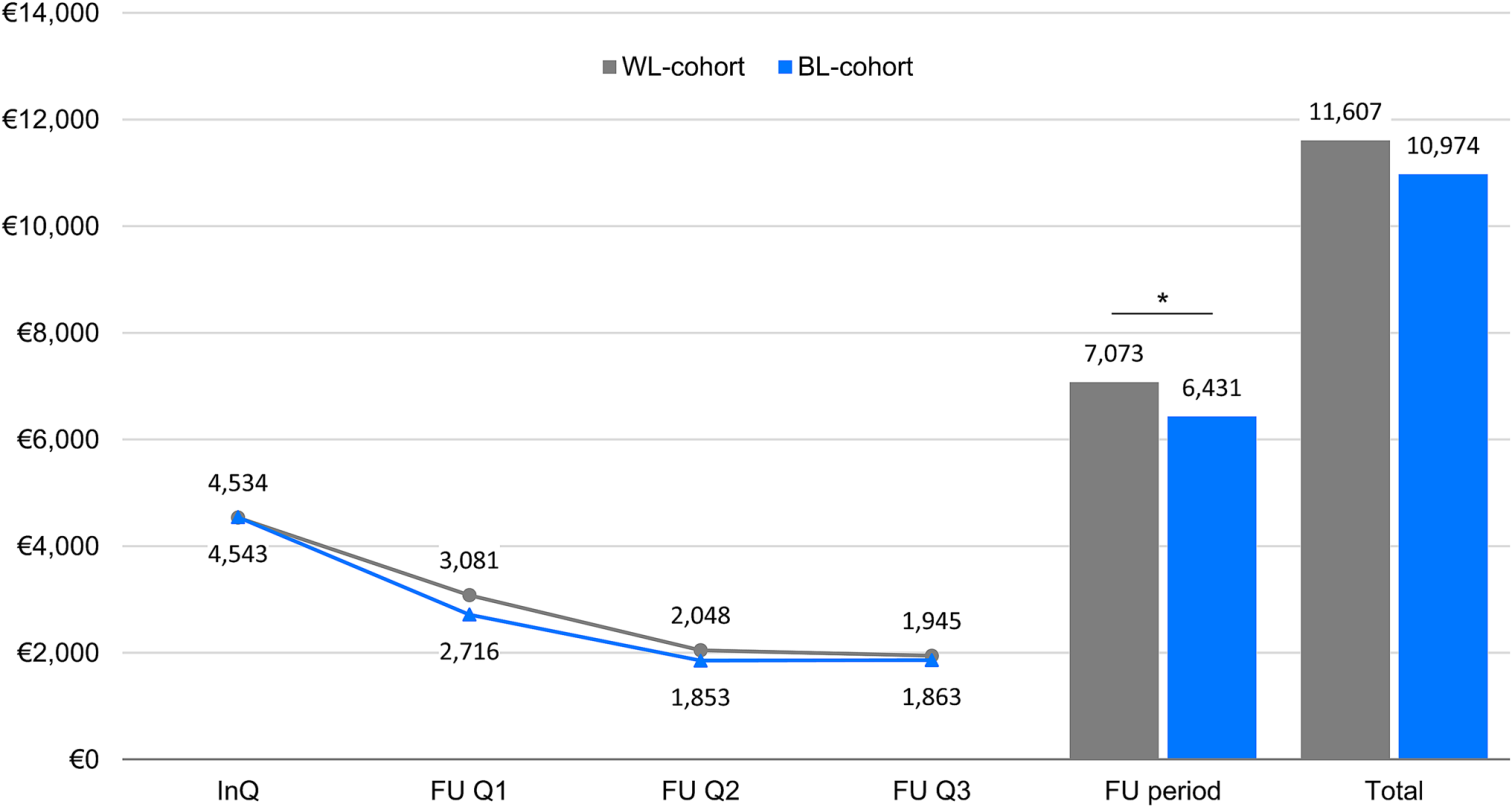
Total costs and development of costs per cohort in the index quarter and follow-up period (adjusted study population). Data are given as mean. WL- vs. BL-cohort *p < 0.05. FU, follow-up period (3 quarters); InQ, index quarter; Total (index quarter + 3 quarters follow-up)

The adjusted study population comprised 4541 patients, with 3582 (78.9%) in the WL- and 959 (21.1%) in the BL-cohort. In accordance with the first results of the subgroup analyses without cystectomy (Table 2B), mean total costs in the InQ were comparable in both cohorts (BL: EU 4543 vs WL: EUR 4534). Over the entire three FU quarters, the mean total costs of EUR 6431 in the BL-cohort were significantly lower (*p* = 0.045) than the mean total costs of EUR 7073 in the WL-cohort (Fig. 2).

Regarding median values, total costs in the InQ were slightly higher in the BL-cohort (EUR 3704 vs EUR 3320). However, over the FU period, also the median total costs of EUR 4426 in the BL-cohort were below the total of the WL-cohort (EUR 4655), thereby “compensating” the higher median initial costs in the BL-cohort.

Considering the individual quarters, the mean total costs in each FU quarter were clearly lower as in the InQ in both cohorts. In each FU quarter, the quarterly costs tended to be lower in the BL- than in the WL-cohort; those differences were most prominent in the second FU quarter but decreased in the third FU quarter. After one year a comparable cost level was reached (Fig. 2).

### Subsequent cystoscopies/TURB of the adjusted study population

After the initial TURB, most patients of the BL-cohort and the WL-cohort were subsequently examined by at least one cystoscopy in InQ and FU. Regarding TURB, in total a higher proportion of patients was subsequently examined by WL-TURB than by BL-TURB in both cohorts. The rate of subsequent WL-TURB was significantly higher in the WL-cohort than in the BL-cohort. A significantly higher proportion of the BL-cohort than of the WL-cohort subsequently received further BL-TURB (*data not shown*).

## Discussion

This comparative retrospective analysis of real-world data on prevailing TURB treatments of BCa showed that in Germany only 20% of GKV-insured persons with BCa receive initial BL-TURB, despite its therapeutic benefits compared to WL-TURB and its recommendation in guidelines [3, 25]. Availability of equipment and acquisition costs as well as additional costs of HAL may restrict the use of BL-TURB in clinical practice. Furthermore, a bias due to incorrect coding of surgical procedures with a possible impact on the unbalanced distribution ratio cannot be excluded.

In this study, initial BL-TURB resulted only in the primary study population in higher initial costs compared to WL-TURB. Subgroup analysis identified cystectomy as a main contributor to costs, masking potential directly TURB-mediated differences. When cystectomy was excluded for further analyses in an adjusted study population, the initial mean total costs of BL-TURB were comparable to WL-TURB, and in the FU, costs of BL-TURB even tended to be lower compared to WL-TURB until they equalized after one year. The view on the median costs supports the trend of the superiority of BL-TURB compared to WL-TURB regarding total costs in the first year: Albeit the initial median costs of the BL-cohort tended to be higher, they were compensated by the lower mean FU costs. The findings of this study are in line with previous results of models for the cost-utility analysis of BL-TURB. Here, higher or similar initial costs of BL-TURB compared to WL-TURB are predicted which are compensated by cost benefits in the long term due to improved patient outcomes [20–23]. In a Markov model calculated for Germany, additional initial BL-TURB reduced costs by EUR 537 per patient compared to only WL-TURB. At the same time, quality-adjusted life years were increased [22]. Witjes et al. [20] suggest that previous restrictions of BL-TURB due to budget need to be adjusted to recent long-term follow-up data and cost analyses.

The study specifically focused on real-world BCa-treatment costs of initial WL- versus BL-TURB in Germany based on claims data, whose original function is reimbursement of healthcare costs. Therefore, the study results were dependent on the quality of coding and classification and apply for German statutory health insurances only. Outcomes irrelevant for reimbursement may be precluded, resulting in under-representation or inadequately documentation of clinical factors like metastases.

The analyzed claims data do not allow the confirmation of the medical hypothesis that BL-TURB is associated with a higher risk reduction of recurrence than WL-TURB. The design of this study involved a pre-index period without TURB or cystectomy and concurrent diagnosis of BCa to ensure that only patients with their initial TURB in the selection period were included. However, this approach does not exclude subsequent TURB completely. Furthermore, low and varying numbers of patients in the subgroup analysis may impact the respective results. Unfortunately, the retrospective study feature does not allow to evaluate the assignment reason to the two procedures WL or BL which is one aspect of the main limitation of this healthcare study: the lack of clinical parameters.

## Conclusion

This comparative retrospective analysis of healthcare claims data provides information on real-world costs of standard WL- or HAL-guided BL-TURB for treatment of BCa in Germany. The application of a BL-TURB does not imply higher initial and consecutive costs than the WL-TURB. In combination with a higher tumor detection rate and consequently lower recurrence risk, described in a variety of clinical trials and publications, BL-TURB constitutes a valuable addition to standard WL-TURB. Nonetheless, initial BL-TURB was still only performed in every fifth GKV-insured patient with BCa. However, limitations of the analysis of healthcare claims data need to be considered, when interpreting the study results.

## Footnotes

1 Total costs considered the following cost domains: outpatient, inpatient, medicinal products, appliances, remedies, and sick pay.
